# Autoimmune cytopenias in inborn errors of immunity: associations with monogenic mutations and immunologic parameters

**DOI:** 10.1186/s12865-025-00752-1

**Published:** 2025-09-24

**Authors:** Ferhat Sağun, Fatih Çölkesen, Mehmet Emin Gerek, Şükran Aslan Savaş, Seçim Kolak, Emrah Harman, Şevket Arslan

**Affiliations:** Division of Clinical Immunology and Allergy, Department of Internal Medicine, Necmettin Erbakan University Faculty of Medicine, Konya, Turkey

## Abstract

**Background:**

Autoimmune cytopenias (AICs) are among the most frequent non-infectious complications in inborn errors of immunity (IEIs) and may represent early or even initial manifestations. The genetic underpinnings of AICs in IEIs remain heterogeneous and incompletely defined.

**Objective:**

This study aimed to determine the prevalence and distribution of AICs and to investigate their associations with underlying monogenic mutations and selected immunophenotypic parameters in adult patients with IEI.

**Methods:**

A total of 121 adult IEI patients from a single tertiary immunology center were evaluated retrospectively. Clinical, immunophenotypic, and genetic data were obtained from electronic medical records. Comparisons were made between patients with and without autoimmune manifestations and AICs. Monogenic mutations were identified using targeted next-generation sequencing (NGS).

**Results:**

Autoimmune manifestations were present in 48 of 121 patients (39.6%), and autoimmune cytopenias (AICs) were identified in 33 patients (27.5%). Autoimmune hemolytic anemia (AIHA) was the most frequently observed subtype, followed by combined cytopenias and immune thrombocytopenia (ITP). The most common genetic alteration detected was a mutation in *TNFRSF13B* (TACI), with additional variants identified in *DOCK8*, *RAG1*, *LRBA*, *PRF1*, *PSTPIP1*, *CECR1*, *PRKDC*, and *MRTFA*. Logistic regression revealed a strong independent association between TACI mutations and ITP (OR: 46.5, *p* = 0.002), while no significant relationship was found with autoimmune cytopenias overall. No statistically significant differences were found in class-switched memory B cells (CD27⁺IgD⁻) percentages, CD4⁺/CD8⁺ T-cell ratios, or baseline IgG concentrations between patients with and without autoimmune manifestations or AICs.

**Conclusion:**

AICs represent a significant clinical burden in adult IEIs and may occur in association with a wide range of genetic variants. Class-switched memory B cells (CD27⁺IgD⁻) percentages, CD4⁺/CD8⁺ T-cell ratio, and baseline IgG were not significantly associated with autoimmunity in this cohort. These findings underscore the need for broader immunophenotypic and genetic screening to improve the early recognition and management of autoimmune complications in IEIs.

## Introduction

Inborn errors of immunity (IEIs) are a group of disorders that are characterized by increased susceptibility to infections, malignancies, immune dysregulation, and autoimmunity complications [1]. Autoimmune manifestations are often the first, or sometimes the only, clinical signs of immune dysregulation in certain patients with IEIs [2]. Among autoimmune complications, autoimmune cytopenias (AICs)—including autoimmune hemolytic anemia (AIHA), immune thrombocytopenia (ITP), autoimmune neutropenia (AIN), and Evans syndrome—are the most common and clinically significant presentations in both pediatric and adult IEI populations [3, 4]. Fischer et al. reported that 26% of 2,183 patients in the French national IEI cohort had immune dysregulatory features, and AICs were observed in 31.4% of those 571 patients [5]. In an Italian single-center study, AICs were observed in 17.8% of 95 adult Common Variable Immunodeficiency (CVID) patients, with ITP being the most common (10.5%) [6]. In a pediatric cohort, 17 out of 154 patients with AICs were diagnosed with IEI, with AIHA and Evans syndrome being more frequent among them [7]. A systematic review of 31 studies involving 3,991 CVID patients reported a pooled prevalence of hematologic autoimmunity at 18.9%, including ITP, AIHA, AIN, and Evans syndrome [8].

AICs significantly contribute to the disease burden in IEI patients, often requiring long-term immunosuppressive therapies and hospitalization. Although AICs may respond to corticosteroids or intravenous immunoglobulin (IVIG), many patients suffer from refractory or relapsing disease courses [4]. This resistance to treatment is often linked to underlying monogenic defects in genes that regulate T and B cell tolerance, along with immune checkpoint signaling [9, 10]. Pathogenic variants in CTLA4, LRBA, TNFRSF13B (TACI), DOCK8, RAG1, PRF1, PIK3CD, IKZF1, and PRKDC have been identified in patients with AICs and PIDs, often associated with other clinical features such as lymphoproliferation, splenomegaly, and immunologic abnormalities such as hypogammaglobulinemia [10–12]. These genetic findings have therapeutic implications, as targeted immunosuppressive treatments, such as abatacept, rituximab, sirolimus, or mycophenolate mofetil, have shown increased effectiveness in patients with defined immune dysregulation syndromes [4, 10, 11, 13, 14]. Although AICs in IEIs are gaining recognition, data from adult patient populations remain limited. Further studies are required to better define their prevalence, underlying genetic causes, and clinical impact.

In this study, we conducted a retrospective evaluation of adult patients diagnosed with IEI at a single tertiary immunology center to determine the frequency and distribution of AICs and to identify the spectrum of underlying monogenic mutations. By analyzing the clinical, immunophenotypic, and genetic characteristics of these patients, we aimed to gain a clearer understanding of the mechanisms of immune dysregulation contributing to cytopenias and to highlight the importance of early genetic evaluation. We also examined whether immunological parameters—such as the frequency of class-switched memory B cells (CD27⁺IgD⁻), CD4⁺/CD8⁺ T-cell ratio, and baseline serum IgG levels—differed according to the presence of autoimmune manifestations or AICs, to assess their potential relevance in immune dysregulation.

## Materials and methods

### Study design

This retrospective study was conducted at the Division of Clinical Immunology and Allergy, Department of Internal Medicine, Necmettin Erbakan University Faculty of Medicine, Konya, Turkey. Records of 121 adult patients diagnosed with IEI were reviewed from January 2020 to January 2025. The diagnosis of IEIs was based on the criteria defined by the European Society for Immunodeficiencies (ESID) [15]. Patients younger than 18 years or those with secondary immunodeficiencies were excluded.

### Data collection

Demographic characteristics, clinical findings, laboratory results, and autoimmune manifestations were gathered from patient records. Immunologic parameters were considered based on the treatment-naive laboratory values available at the time of diagnosis or at the patient’s first evaluation in our clinic. Class-switched memory B cells (CD27⁺IgD⁻) were measured by flow cytometry and reported as a percentage of B cells. AICs were defined as ITP, AIHA, AIN, Evans syndrome, and other combinations of autoimmune cytopenias. Diagnoses were established based on clinical presentation and hematological assessments, including complete blood count, direct Coombs test, and exclusion of secondary causes. Autoimmune manifestations were defined as the presence of documented autoimmune diseases other than cytopenias, diagnosed by relevant specialists during routine clinical care. These included classical autoantibody-mediated disorders such as connective tissue diseases, autoimmune thyroiditis, autoimmune endocrinopathies, and neurological autoimmune disorders, as well as immune-mediated inflammatory diseases, such as inflammatory bowel disease, which are commonly observed in the context of inborn errors of immunity. Diagnoses were based on clinical and/or laboratory criteria available at the time of documentation and were not re-evaluated retrospectively using current classification guidelines.

Genetic test results from 68 of the 121 patients—regardless of autoimmune cytopenia status—were retrospectively evaluated. Targeted next-generation sequencing (NGS) with clinically relevant IEI gene panels was used to identify monogenic defects. Variants were classified according to ACMG guidelines, and both pathogenic/likely pathogenic variants and variants of uncertain significance (VUS) were documented [16].

### Statistical analysis

Descriptive statistics were used to summarize demographic and clinical characteristics. Categorical variables were expressed as frequencies and percentages, and compared using the Chi-square or Fisher’s exact test, as appropriate. Continuous variables were assessed for normality using the Shapiro–Wilk test. Variables not normally distributed were presented as medians with interquartile ranges (IQR) and compared using the Mann–Whitney U test. Normally distributed continuous variables, when applicable, were expressed as means ± standard deviation and compared using the independent samples t-test. A p-value of < 0.05 was considered statistically significant. All analyses were performed using IBM SPSS Statistics, version 20.

## Results

**Fig. 1:**
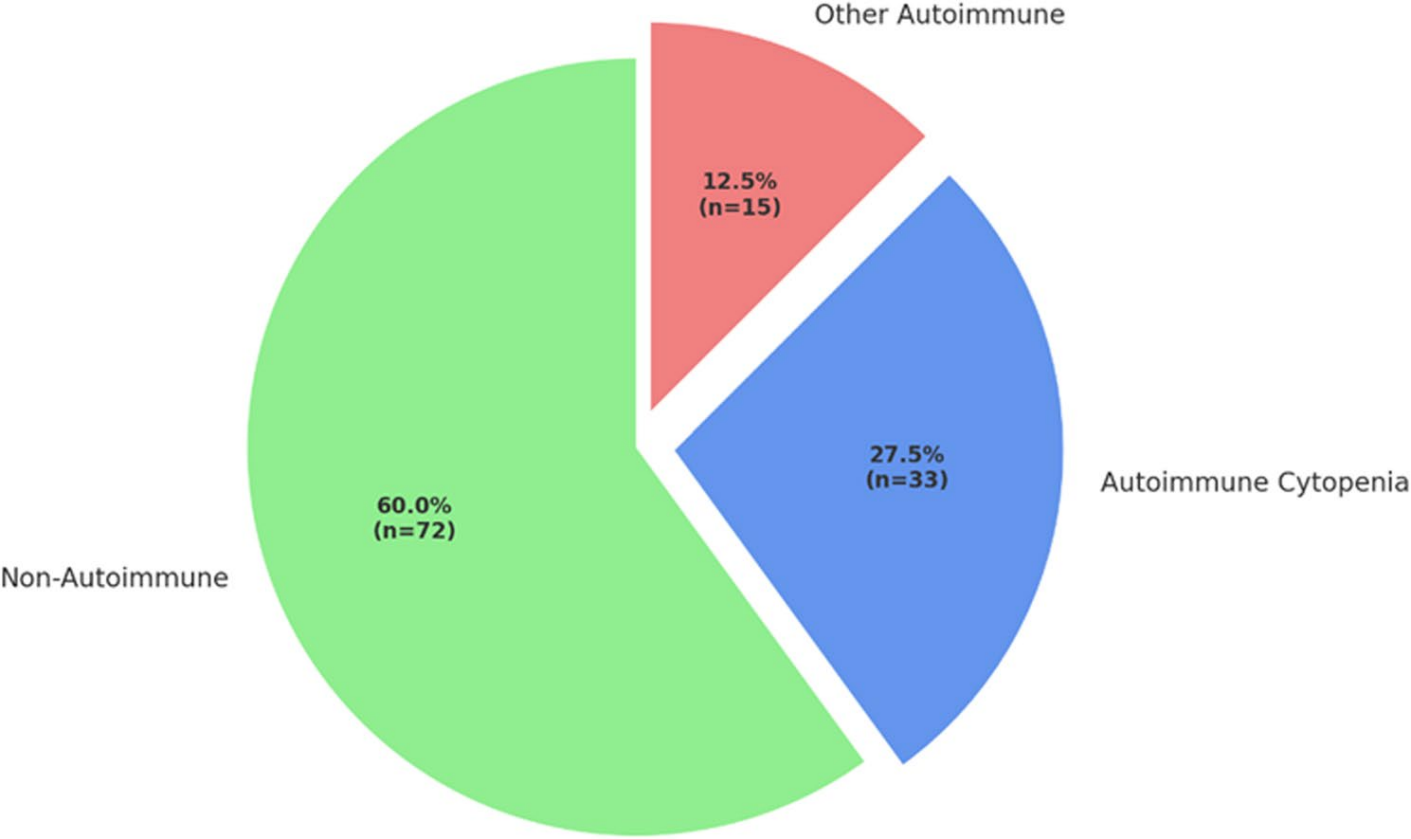
Percentages of autoimmunity and AICs in IEI patients

The median age of the patients was 37 (19–76) years, and 49.6% (*n* = 60) were female. Among the study group, 39.6% (*n* = 48) had at least one autoimmune disease. AICs were detected in 27.5% (*n* = 33) of the patients (Fig. 1). Among the 33 patients with autoimmune cytopenias, 13 had available genetic results. All variants were heterozygous; except for TACI, they were located in genes with autosomal recessive inheritance. The most frequently identified pathogenic variants associated with AICs were found in the TNFRSF13B (TACI) gene. Other pathogenic variants were identified in genes including DOCK8, RAG1, LRBA, PRF1, PSTPIP1, PRKDC, CECR1, and MRTFA (Table 1). Additionally, TACI variants were also identified in 7 of the 55 genetically tested patients without autoimmune cytopenias.

**Table 1:** Genetic variants and diagnoses detected in IEI patients

Patient (*P*)	Sex		Gene	HGVS coding	Variant type	HGVS protein	Variant ID	Autoimmune Cytopenia
P1	M		*TNFRSF13B*	c.310T > C	Missense		rs34557412	ITP
P2	M		*-*	-	-		-	AIHA
P3	M		*-*	-	-		-	Combined autoimmune cytopenia
P4	M		*-*	-	-		-	AIHA
P5	M		*-*	-	-		-	AIHA
P6	M		*-*	-	-		-	Combined autoimmune cytopenia
P7	F		*RAG1*	c.1792G > A	Missense		rs1024345971	Evans Syndrome
P8	M		*LRBA*	c.4522 C > G	Missense	(Q1508E)	-	Evans Syndrome
P9	M		*TNFRSF13B*	c.515G > A	Missense	(C172Y)	rs751216929	ITP
P10	F		*-*	-	-		-	AIHA
P11	M		*DOCK8*	c.3212T > C	Missense	(L1071P)	rs1451278431	AIHA
P12	M		*-*	-	-		-	ITP
P13	M		*-*	-	-		-	Evans Syndrome
P14	M		*-*	-	-		-	Evans Syndrome
P15	M		*DOCK8*	c.1072 A > G	Missense	(I358V)	rs757929543	AIHA
P16	M		*-*	-	-		-	AIHA
P17	M		*-*	-	-		-	AIHA
P18	M		*PSTPIP1*	c.1213 C > T	Missense	(R405C)	rs201253322	AIHA
P19	F		*-*					AIHA
P20	F		*PRF1*	c.1067G > A	Missense	(R356Q)	rs571015630	Evans Syndrome
P21	F		*-*	-	-		-	Combined autoimmune cytopenia
P22	M		*-*	-	-		-	AIHA
P23	F		*TNFRSF13B*	c.452 C > T	Missense	(P151L)	rs200037919	ITP
P24	M		*-*	-	-		-	ITP
P25	F		*CECR1 (ADA2)*	c.144delG	Deletion		rs756881285	AIHA
P26	F		*TNFRSF13B*	c.310T > C	Missense		rs34557412	ITP
P27	F		*-*	-	-		-	Pernicious anemia
P28	M		*-*	-	-		-	Combined autoimmune cytopenia
P29	M		*MRTFA*	c.846 C > G	Missense	(H282Q)	rs372255666	AIHA
P30	F		*PRKDC*	c.1655 C > G	Missense		rs373088662	Combined autoimmune cytopenia
P31	F		*-*	-	-		-	Combined autoimmune cytopenia
P32	F		*-*	-	-		-	Combined autoimmune cytopenia
P33	F		*-*	-	-		-	AIHA

*M* Male, *F* Female, *ITP* Immune Thrombocytopenıc Purpura, *AIHA* Autoımmune Hemolytıc Anemıa, *HGVS* Human Genome Variation Society, coding and protein changes are presented using HGVS nomenclature

**Fig. 2:**
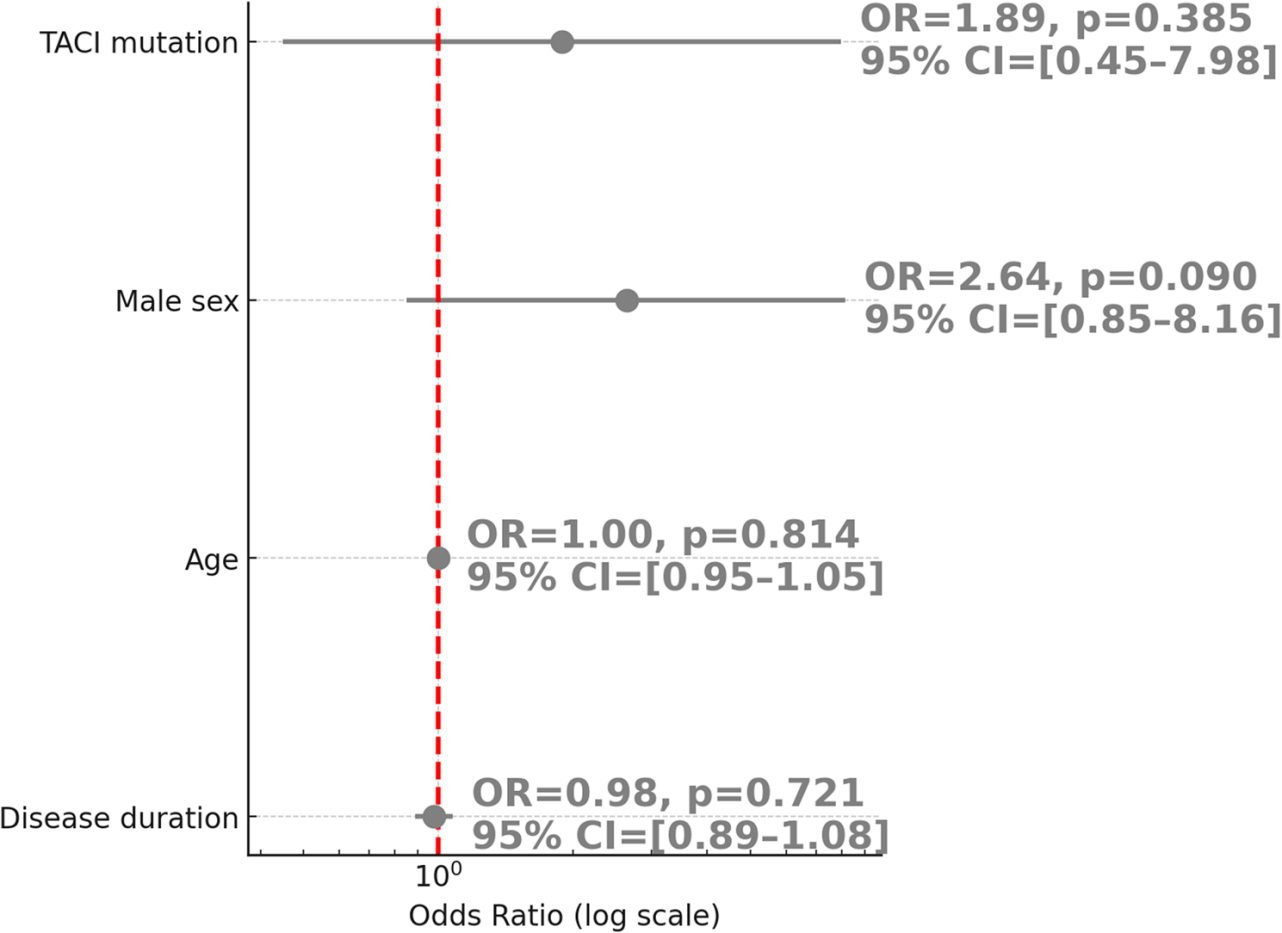
Association Between *TACI* Mutation and Autoimmune Cytopenias. Forest plot showing odds ratios (OR) and 95% confidence intervals (CI) from logistic regression analysis in IEI patients (n = 68). Variables included TACI mutation, sex, age, and disease duration

To investigate the relationship between AICs and the most frequently detected monogenic defect in our cohort, we performed a binary logistic regression analysis evaluating the association between *TACI* mutation and the presence of AIC. Among the 68 patients who underwent genetic testing, the *TACI* mutation was not significantly associated with AIC (OR = 1.89, 95% CI: 0.45–7.98, *p* = 0.385) (Table 2 and Fig. 2).

**Table 2:** Logistic regression analysis of the association between *TACI* mutation and autoimmune cytopenias in IEI patients

Variable	OR (Exp(B))	95% CI (lower–upper)	*p*-value
*TACI* mutation	1.89	0.45–7.98	0.385
Male sex	2.64	0.85–8.16	0.090
Age	1.00	0.95–1.05	0.814
Disease duration	0.98	0.89–1.08	0.721

Binary logistic regression model assessing predictors of autoimmune cytopenias in IEI patients with available genetic testing results (*n* = 68). Results are presented as odds ratios (OR) with corresponding 95% confidence intervals (CI) and *p*-values

*IEI* Inborn errors of immunity

**Fig. 3:**
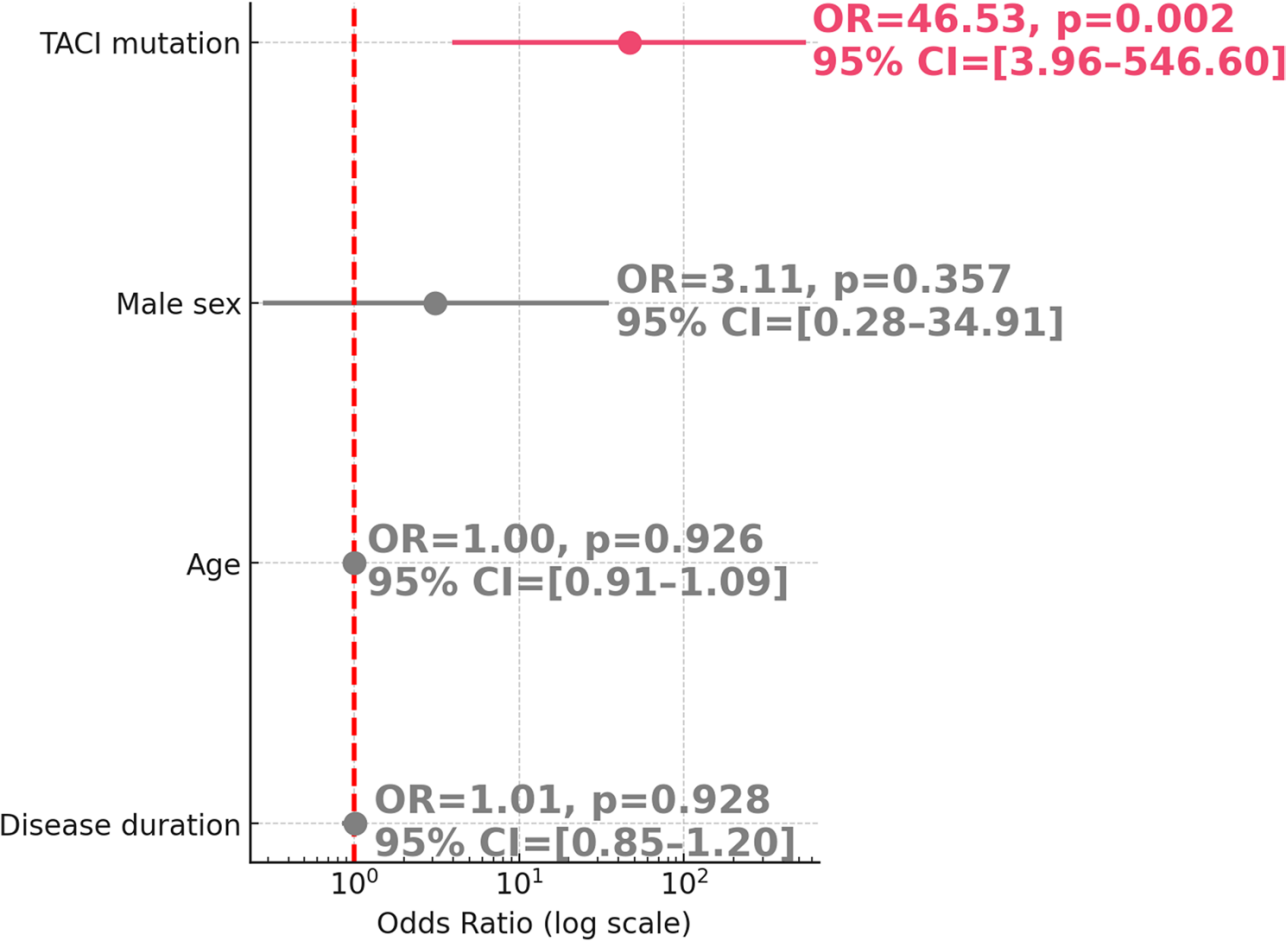
Association Between *TACI *Mutation and ITP. Forest plot showing odds ratios (OR) and 95% confidence intervals (CI) for the association between TACI mutation and ITP in genetically tested IEI patients (*n* = 68). Variables included TACI mutation, sex, age, and disease duration

Given the potential subtype-specific associations, we further analyzed the relationship between *TACI* mutation and ITP. In the same subgroup of genetically tested patients, *TACI* mutation was found to be independently associated with ITP (OR = 46.53, 95% CI: 3.96–546.60, *p* = 0.002), even after adjusting for age, sex, and disease duration (Table 3 and Fig. 3).

**Table 3:** Logistic regression analysis of the association between *TACI* mutation and ITP in genetically tested IEI patients

Variable	OR (Exp(B))	95% CI (lower–upper)	*p*-value
*TACI* mutation	46.53	3.96–546.60	0.002
Male sex	3.11	0.28–34.91	0.357
Age	1.00	0.91–1.09	0.926
Disease duration	1.01	0.85–1.20	0.928

Binary logistic regression model assessing the association between TACI mutation and ITP in IEI patients with available genetic testing results (*n* = 68). Independent variables included TACI mutation status, sex, age, and disease duration. Results are presented as odds ratios (OR) with corresponding 95% confidence intervals (CI) and *p*-values

*IEI* Inborn errors of immunity, *ITP* Immune thrombocytopenia

**Fig. 4:**
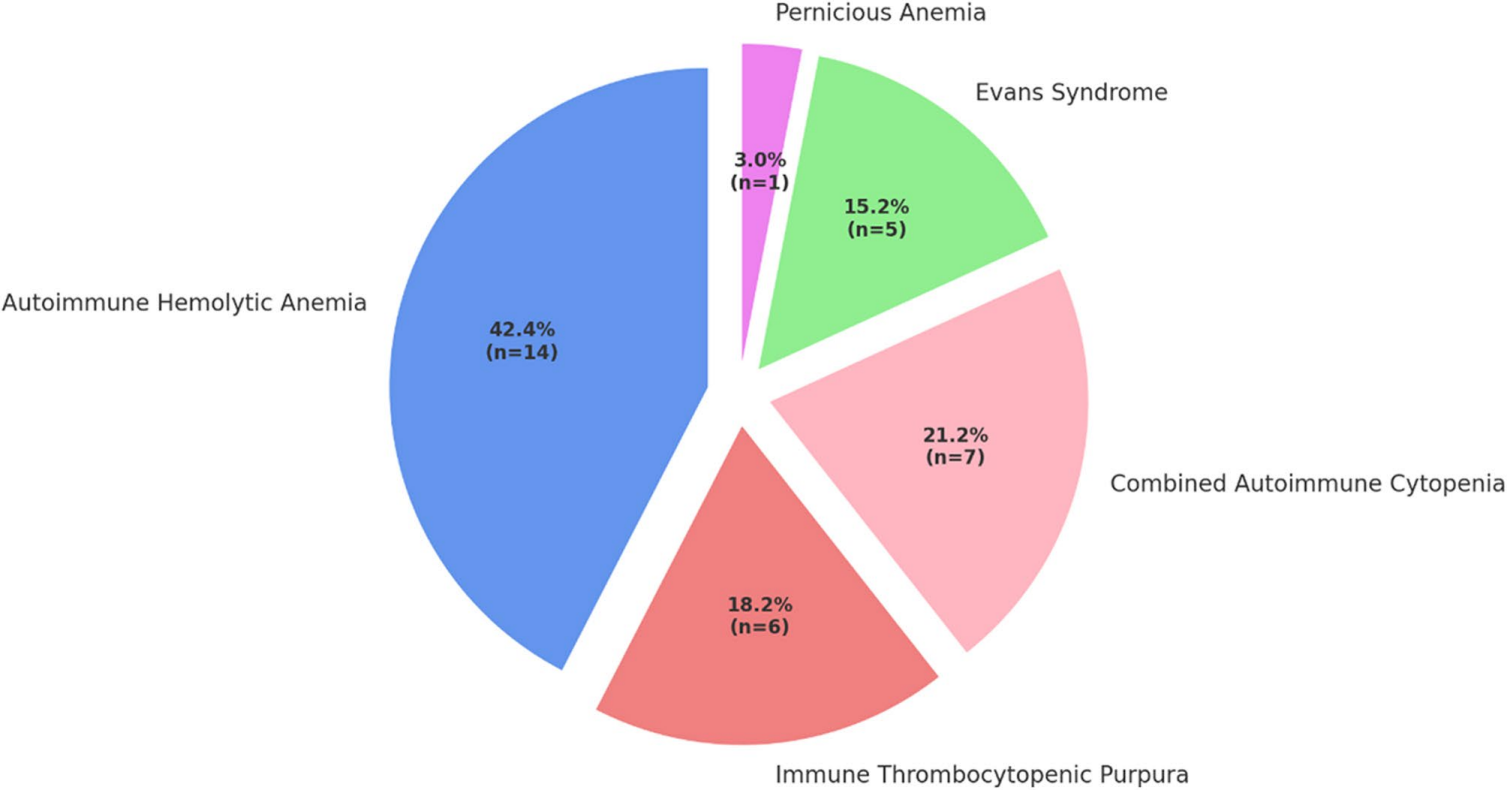
Distribution of Autoimmune Cytopenia Subtypes in IEI Patients Diagnosed with AICs

AIHA was the most common subtype, accounting for (42.4% *n* = 14) of all cases, followed by combined autoimmune cytopenia (21.2%, *n* = 7), ITP (18.2%, *n* = 6), Evans syndrome (15.2%, *n* = 5), and pernicious anemia (3.0%, *n* = 1) (Fig. 4).

**Fig. 5:**
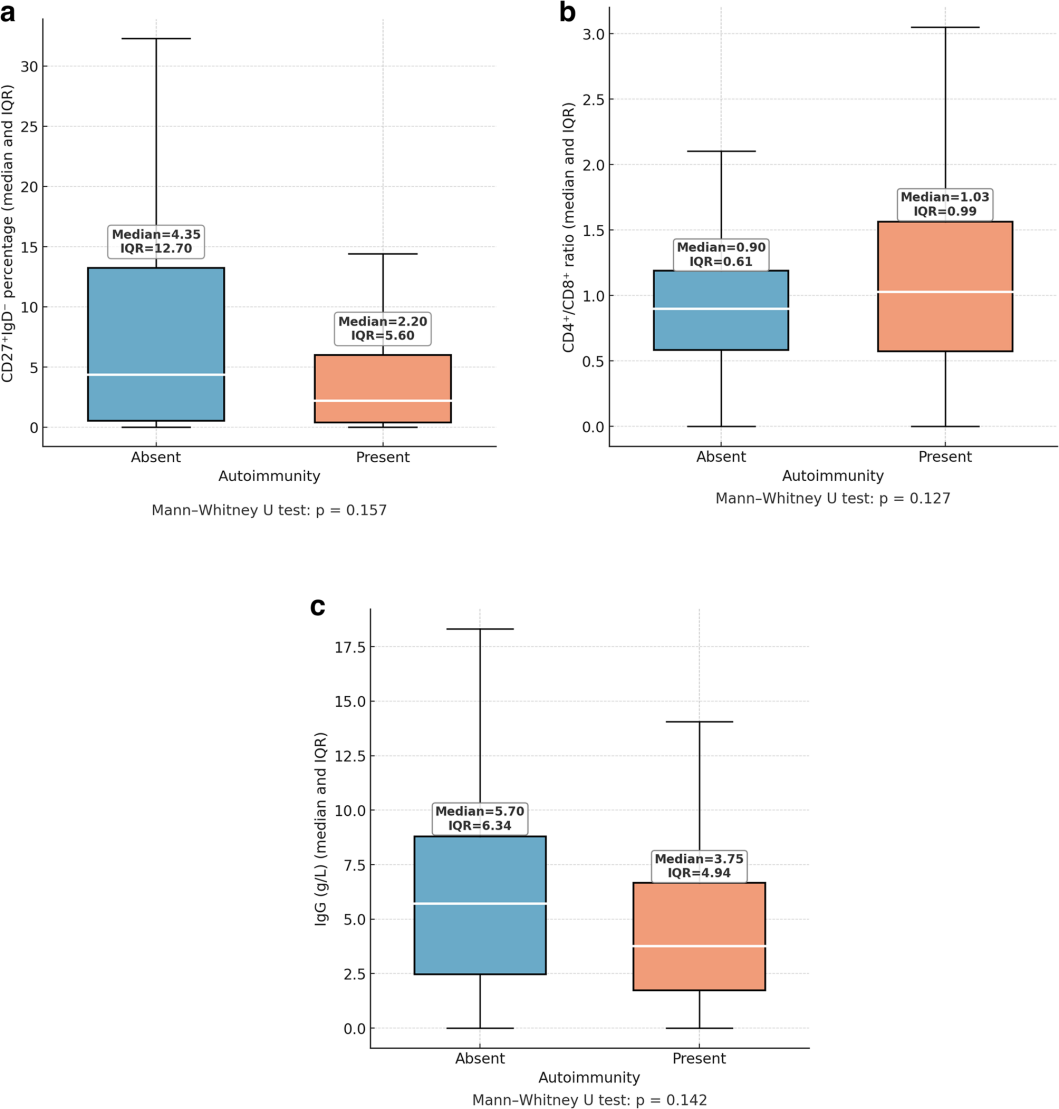
**a **Class-switched memory B cells (CD27⁺IgD⁻) percentages in patients with and without autoimmune manifestations. Values are shown as median and IQR. Median values were lower in the autoimmunity group (2.20%) compared to the non-autoimmunity group (4.35%), but the difference was not significant (*p* = 0.157). **b**. CD4⁺/CD8⁺ T-cell ratio in patients with and without autoimmune manifestations. Values are shown as median and IQR. The difference was not statistically significant (*p* = 0.127). **c**. Baseline serum IgG levels in patients with and without autoimmune manifestations. Values are shown as median and IQR. No significant difference was detected (*p* = 0.142)

**Fig. 6:**
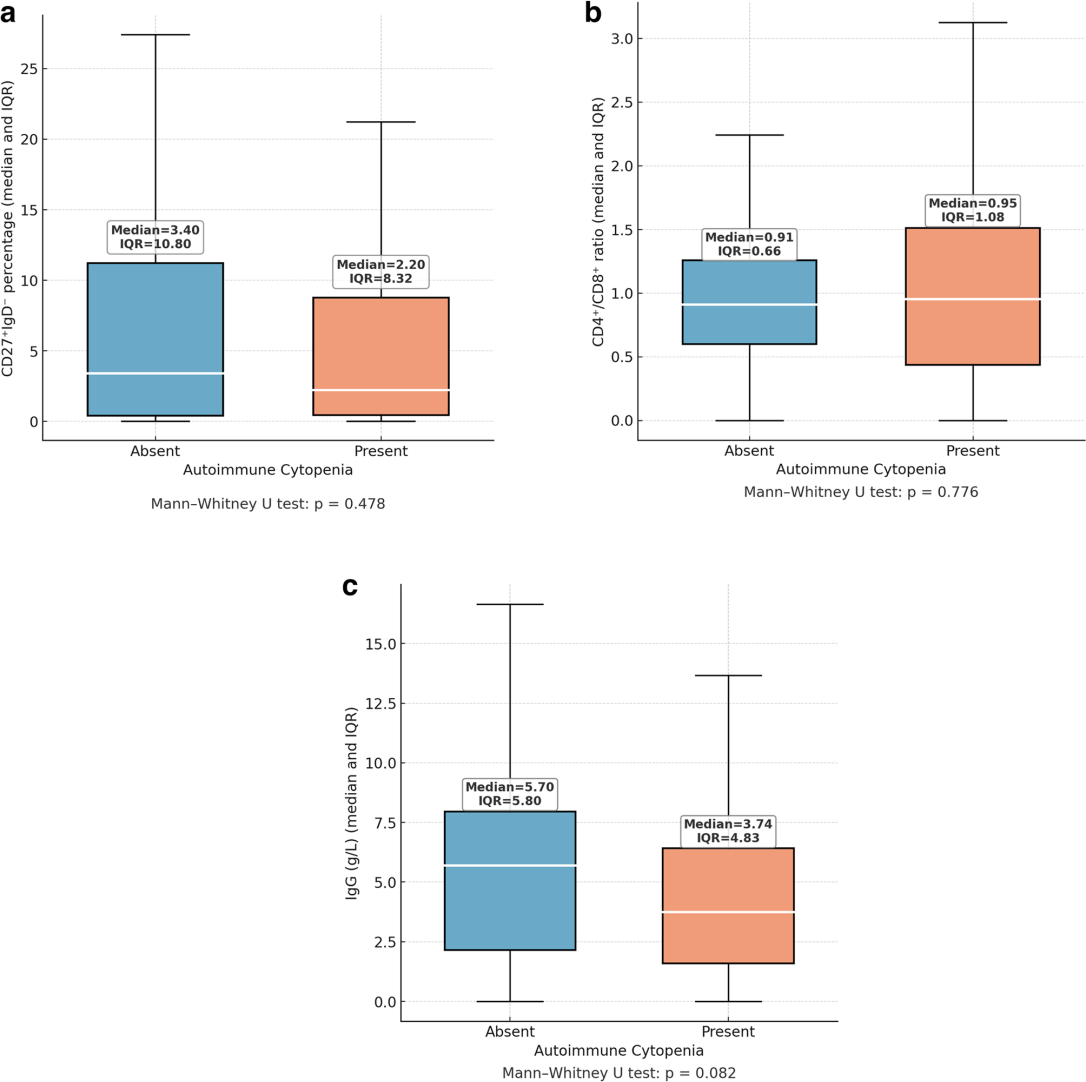
**a **Comparison of class-switched memory B cells (CD27⁺IgD⁻) percentages between patients with and without autoimmune cytopenia. Data are shown as median and interquartile range (IQR). No significant difference was observed (*p* = 0.478). **b**. CD4⁺/CD8⁺ T-cell ratio in patients with and without autoimmune cytopenia. Values are presented as median and IQR. No statistically significant difference was found (*p* = 0.776). **c**. Baseline IgG levels in patients with and without autoimmune cytopenia. Values are shown as median and IQR. The difference did not reach statistical significance (*p* = 0.082)

Class-switched memory B cell percentages (CD27⁺IgD⁻) were compared according to autoimmune manifestations and autoimmune cytopenias. In patients without autoimmune manifestations, the median percentage was 4.35% (IQR: 0.53–13.23), while in those with autoimmune manifestations it was 2.20% (IQR: 0.40–6.00), with no statistically significant difference (*p* = 0.157). Similarly, patients without autoimmune cytopenias had a median CD27⁺IgD⁻ percentage of 3.40% (IQR: 0.40–11.20), whereas those with autoimmune cytopenias had 2.20% (IQR: 0.43–8.75) (*p* = 0.478) (Table 4, Figs. 5a and 6a)

**Table 4:** Comparison of Class-Switched memory B cell percentages (CD27⁺IgD⁻) in IEI patients according to the presence of autoimmune manifestations and autoimmune cytopenias

Group	*n*	Median (IQR)	*p*-value
Autoimmune Manifestation			
Absent	60	4.35 (0.53–13.23)	0.157
Present	47	2.20 (0.40–6.00)	
Autoimmune Cytopenia (AIC)			
Absent	75	3.40 (0.40–11.20)	0.478
Present	32	2.20 (0.43–8.75)	

Data are presented as median and interquartile range (IQR) due to non-normal distribution. Statistical comparisons were conducted using the Mann–Whitney U test. No statistically significant differences were observed between groups. CD27⁺IgD⁻ cells were assessed by flow cytometry and expressed as a percentage of total B cells

*IEI* Inborn Errors of Immunity

The CD4⁺/CD8⁺ T-cell ratio was also analyzed in relation to autoimmune features. Patients without autoimmune manifestations had a median CD4⁺/CD8⁺ T-cell ratio of 0.90 (IQR: 0.58–1.19), compared to 1.03 (IQR: 0.58–1.56) in those with autoimmune manifestations (*p* = 0.127). When assessed by autoimmune cytopenia status, the median CD4⁺/CD8⁺ T-cell ratio was 0.91 (IQR: 0.60–1.26) in patients without cytopenias and 0.95 (IQR: 0.44–1.51) in those with cytopenias (*p* = 0.776) (Table 5, Figs. 5b and 6b).

**Table 5:** Comparison of CD4⁺/CD8⁺ ratios in IEI patients according to the presence of autoimmune manifestations and autoimmune cytopenias

Group	*n*	Median (IQR)	*p*-value
Autoimmune Manifestation			
Absent	72	0.90 (0.58–1.19)	0.127
Present	47	1.03 (0.58–1.56)	
Autoimmune Cytopenia (AIC)			
Absent	87	0.91 (0.60–1.26)	0.776
Present	32	0.95 (0.46–1.51)	

CD4⁺/CD8⁺ T cell ratios are expressed as median and interquartile range (25th–75th percentile) due to non-normal distribution. Comparisons were performed using the Mann–Whitney U test

*IEI* Inborn Errors of Immunity

Baseline IgG levels were compared between patients with and without autoimmune manifestations and autoimmune cytopenias. The median IgG level was 5.70 g/L (IQR: 2.46–8.80) in patients without autoimmune manifestations and 3.76 g/L (IQR: 1.72–6.66) in those with, showing no statistically significant difference (*p* = 0.142). Similarly, patients with autoimmune cytopenias had a median IgG level of 3.74 g/L (IQR: 1.59–6.42), compared to 5.70 g/L (IQR: 2.15–7.95) in those without, which also did not reach statistical significance (*p* = 0.082) (Table 6, Figs. 5c and 6c).

**Table 6:** Comparison of serum IgG levels (g/L) in IEI patients according to the presence of autoimmune manifestations and autoimmune cytopenias

Group	*n*	Median (IQR)	*p*-value
Autoimmune Manifestation			
Absent	71	5.70 (2.46–8.80)	0.142
Present	48	3.75 (1.72–6.66)	
Autoimmune Cytopenia (AIC)			
Absent	86	5.70 (2.15–7.95)	0.082
Present	33	3.74 (1.59–6.42)	

Serum IgG levels are expressed as median and interquartile range (25th–75th percentile) due to non-normal distribution. Comparisons were performed using the Mann–Whitney U test

*IEI *Inborn Errors of Immunity

## Discussion

This study summarizes the key findings regarding AICs and monogenic mutations in adult patients with IEI. Autoimmune manifestations were observed in 39.6%, and AICs in 27.5% of cases, with AIHA being the most frequent subtype. The most common genetic variant was *TNFRSF13B (TACI)*, followed by *DOCK8*,* RAG1*,* LRBA*,* PRF1*,* PSTPIP1*,* CECR1*,* PRKDC*, and *MRTFA* mutations. No statistically significant differences were found in class-switched memory B cells (CD27⁺IgD⁻) percentages, CD4⁺/CD8⁺ T-cell ratios, or baseline IgG levels between patients with and without autoimmune features. These findings underscore the clinical and immunogenetic heterogeneity characteristic of IEIs and may enhance our understanding of autoimmune complications within this patient population.

Autoimmune manifestations are increasingly recognized as common features in patients with IEI, often serving as early or even the initial clinical presentation [17]. Several extensive cohort studies have reported autoimmune involvement in approximately 30% of IEI patients, with higher rates observed in specific subtypes such as CVID [5, 8]. Notably, AICs are among the most frequent and clinically significant autoimmune complications. In our cohort, autoimmune manifestations were present in 39.6% of patients, while AICs were detected in 27.5%. This finding aligns with the French national IEIs cohort, which reported that among 2,183 IEI patients, 26% had immune dysregulatory features, and 31.4% had AICs [5]. Similarly, a meta-analysis involving 3,991 CVID patients found a pooled prevalence of hematologic autoimmunity at 18.9%, emphasizing the clinical burden of AICs in this population [8]. In this study, AIHA was identified as the most common AIC in patients with IEIs. Combined autoimmune cytopenia and ITP were other common cytopenias. However, ITP is generally reported as IEIs cohorts’ most common AIC [7, 18–20]. In contrast, AIHA was our study’s most frequently observed cytopenia. This difference may be attributed to the single-center nature of our cohort and the relatively small sample size, which could have influenced the distribution of AIC subtypes. Although the relative frequencies of AIC subtypes may vary across different cohorts, these findings highlight the substantial burden of immune dysregulation in adult patients with IEI and underscore the importance of early recognition and classification of autoimmune complications.

The most frequently identified genetic alteration in our cohort was the *TACI* mutation, followed by mutations in *DOCK8*,* RAG1*,* LRBA*,* PRF1*,* PSTPIP1*,* CECR1*,* PRKDC*, and *MRTFA. TACI* mutations are widely recognized as key contributors to immune dysregulation in IEIs, particularly in CVID. According to extensive cohort studies, 7–10% of CVID patients carry heterozygous *TACI* variants, indicating its role as a susceptibility gene or disease modifier [21, 22]. These mutations have been linked to increased rates of autoimmunity and splenomegaly. They impair B cell function and isotype switching, leading to defective germinal center formation and dysregulated antibody production, possibly contributing to AICs [22]. In a Greek national cohort, *TACI* mutations were associated with AICs such as AIHA and ITP, further supporting their relevance to immune dysregulation in CVID [23]. Similarly, an extensive U.S. cohort study identified *TACI* as the most frequent genetic defect and found that autoimmune manifestations—particularly AIHA and ITP—were significantly more common in *TACI*-mutated patients [24]. Matson et al. also reported that CVID patients with *TACI* mutations frequently exhibit expanded populations of CD21^low B cells, a dysregulated B cell subset strongly linked to AICs [25]. Altogether, these data underscore the pivotal role of *TACI* mutations in the pathogenesis of immune dysregulation and AICs within the spectrum of IEIs. To further dissect the clinical implications of these findings, we performed additional statistical analyses focusing on the relationship between genetic mutations and specific AIC subtypes.

Although *TACI* mutations were the most frequently observed genetic alterations in our cohort, logistic regression analysis failed to demonstrate a significant association between *TACI* mutation and the presence of autoimmune cytopenias as a whole. This suggests that *TACI*-related immune dysregulation may not uniformly predispose to all subtypes of cytopenia. However, when explicitly analyzed in patients with ITP, the *TACI* mutation emerged as a strong independent predictor, with nearly 47-fold increased odds of ITP (*p* = 0.002). This finding highlights the importance of evaluating individual autoimmune manifestations separately in genetically heterogeneous IEI populations and may have implications for pathophysiological understanding and clinical management in such subgroups. This divergence may reflect subtype-specific patterns of immune dysregulation in patients with *TACI* mutations. Further studies are needed to clarify whether certain autoimmune cytopenias are more strongly associated with *TACI*-related defects than others.

Due to the low number of patients carrying other monogenic defects such as *LRBA*,* CTLA4*, or *DOCK8* mutations, further regression modeling for their association with autoimmune cytopenias was not feasible in our dataset. Future studies with larger multicenter cohorts are needed to elucidate better genotype–phenotype correlations across a broader spectrum of genetic variants.

In addition to *TACI*, several other monogenic variants identified in our cohort have been implicated in immune dysregulation and hematologic autoimmunity. *DOCK8* deficiency, though classically associated with hyper-IgE syndrome and viral susceptibility, has also been linked to AICs and vasculitic manifestations [26, 27]. *LRBA* deficiency is strongly associated with immune dysregulation and is frequently accompanied by autoimmunity, particularly AICs, due to impaired *CTLA-4* expression and regulatory T cell dysfunction [28]. *PRF1* mutations disrupt cytotoxic lymphocyte function and lead to chronic immune activation, thereby contributing to the pathogenesis of autoimmune disease [29]. *CECR1* mutations, which cause deficiency of adenosine deaminase 2 (DADA2), are associated with cytopenias—including AIHA, thrombocytopenia, and severe neutropenia—that may occasionally be the initial clinical presentation [30]. No published studies have demonstrated a direct association between *PRKDC* or *MRTFA* mutations and AICs. *PSTPIP1* mutations cause cytopenias through autoinflammatory pathways, but are not linked to classical AICs such as AIHA or ITP [31]. Early identification of genetic variants associated with AICs in IEI patients is crucial for initiating appropriate immunomodulatory therapies. Timely diagnosis may reduce morbidity and guide targeted interventions tailored to underlying genetic defects.

Despite the single-center design and limited sample size, our findings appear to be consistent with those of larger registries. The prevalence of autoimmune cytopenias in our cohort (27.5%) aligns with reports from the French national IEIs cohort (31.4%) and the USIDNET registry (28%) [5, 19]. Moreover, the spectrum of genetic variants identified in our patients—including *TNFRSF13B*, *DOCK8*, *RAG1*, and *LRBA*—closely resembles the mutational landscape reported in prior studies of CVID and other antibody deficiencies [21, 22, 24, 26–28]. These parallels support the representativeness of our cohort within the broader context of inborn errors of immunity.

Low baseline IgG levels have been associated with poorer clinical outcomes in IEIs, particularly in CVID, where they correlate with decreased survival [32]. While hypogammaglobulinemia increases the risk of infections, current evidence does not support a direct connection between baseline IgG levels and the development of autoimmune manifestations or cytopenias. Similarly, no significant association was found between baseline IgG levels and autoimmune features or AICs in our cohort. Patients with autoimmune manifestations and AICs in our cohort exhibited lower mean class-switched memory B cells (CD27⁺IgD⁻) percentages than those without such findings; however, the differences were not statistically significant. Multiple studies, including those by Warnatz, Piqueras, and the EUROclass group, consistently demonstrated that a marked reduction in switched memory B cells (CD27⁺IgM⁻IgD⁻) in CVID patients is associated with immune dysregulation features such as splenomegaly, lymphadenopathy, and AICs [33–35]. Similarly, lower levels of isotype-switched memory B cells have been linked to a higher risk of autoimmune manifestations, further underscoring their role as a marker of immune dysregulation in CVID [32, 36, 37]. Studies have shown that patients with CVID and autoimmune manifestations often exhibit altered CD4⁺/CD8⁺ T-cell ratios along with reduced regulatory T cells, indicating that T cell imbalance may contribute to immune dysregulation and the development of autoimmunity [38]. However, no statistically significant difference in CD4⁺/CD8⁺ T-cell ratios was observed in our cohort between patients with autoimmune features and those without them, or between patients with and without AICs. The absence of statistically significant differences in class-switched memory B cells (CD27⁺IgD⁻) percentages and CD4⁺/CD8⁺ T-cell ratios in our study might result from the limited sample size, which diminishes the ability to detect subtle variances. Additionally, the genetic and clinical heterogeneity of IEI disorders can lead to variable immunophenotypic profiles, obscuring consistent patterns and limiting the detection of statistically significant differences.

This study has several limitations. First, its relatively small sample size and single-center, cross-sectional design may reduce statistical power and limit the generalizability of the findings. Second, although key immunophenotypic parameters such as class-switched memory B cells (CD27⁺IgD⁻) and CD4⁺/CD8⁺ T-cell ratios were analyzed, functional immune assays were not included. Third, genetic testing could not be performed in all patients due to limited access, and some results remained inconclusive, restricting a comprehensive evaluation of genotype–phenotype correlations. Additionally, since genetic testing may have been performed selectively based on clinical features rather than applied systematically across the entire cohort, selection bias could have influenced the observed frequency of monogenic variants. Moreover, the targeted gene panel used for sequencing may not have captured all potentially relevant pathogenic variants, which could have led to an underestimation of certain genetic associations.

In conclusion, our study highlights the substantial impact of AICs and monogenic mutations in adult IEI patients. Our findings suggest that *TACI* mutations may predispose individuals to certain autoimmune conditions, particularly ITP, though not to all types of cytopenia. Although no statistically significant associations were found between class-switched memory B cells (CD27⁺IgD⁻) percentages, CD4⁺/CD8⁺ T-cell ratios, or baseline IgG levels and autoimmune manifestations, these trends underscore the complex and heterogeneous nature of immune dysregulation in IEIs. Comprehensive genetic evaluation and immunophenotypic profiling remain essential for early diagnosis, risk stratification, and the implementation of targeted therapies. Future multicenter studies with larger cohorts and functional analyses are warranted to identify reliable biomarkers of autoimmunity in this population.

## Data Availability

All data generated or analyzed during this study are included in this article. The datasets analysed in this study are available in public repositories as follows. Patient-level accession numbers and persistent links are provided below for 12 patients; for one patient (P8; LRBA, ENST00000357115:c.4522 C>G, p.Q1508E), no public database identifier could be identified as of 12 Aug 2025. The authors do not have access to the raw sequencing files and therefore cannot deposit them in a public repository. Further inquiries can be directed to the corresponding author. P1 — TNFRSF13B c.310T>C (p.Cys104Arg)ClinVar: RCV000005623 → https://www.ncbi.nlm.nih.gov/clinvar/RCV000005623/dbSNP: rs34557412 P7 — RAG1 c.1792G>A (p.Val598Leu)ClinVar (Variation): https://www.ncbi.nlm.nih.gov/clinvar/variation/2285175/dbSNP: rs1024345971 P8 — LRBA c.4522 C>G (p.Gln1508Glu)(Public database identifier not available) P9 — TNFRSF13B c.515G>A (p.Cys172Tyr)ClinVar (Variation): https://www.ncbi.nlm.nih.gov/clinvar/variation/449548/dbSNP: rs751216929 P11 — DOCK8 c.3212T>C (p.Leu1071Pro)dbSNP: rs1451278431 → https://www.ncbi.nlm.nih.gov/snp/rs1451278431 P15 — DOCK8 c.1072 A>G (p.Ile358Val)ClinVar (Variation): https://www.ncbi.nlm.nih.gov/clinvar/variation/3085101/dbSNP: rs757929543 P18 — PSTPIP1 c.1213 C>T (p.Arg405Cys)ClinVar (Variation): https://www.ncbi.nlm.nih.gov/clinvar/variation/242307/dbSNP: rs201253322 P20 — PRF1 c.1067G>A (p.Arg356Gln)dbSNP: rs571015630 → https://www.ncbi.nlm.nih.gov/snp/rs571015630 P23 — TNFRSF13B c.452 C>T (p.Pro151Leu)ClinVar (Variation): https://www.ncbi.nlm.nih.gov/clinvar/variation/546790/dbSNP: rs200037919 P25 — CECR1/ADA2 c.144delG (p.Arg49fs)ClinVar (Variation): https://www.ncbi.nlm.nih.gov/clinvar/variation/640066/dbSNP: rs756881285 P26 — TNFRSF13B c.310T>C (p.Cys104Arg)ClinVar (Variation): https://www.ncbi.nlm.nih.gov/clinvar/variation/5302/dbSNP: rs34557412 P29 — MRTFA c.846 C>G (p.His282Gln)ClinVar (Variation): https://www.ncbi.nlm.nih.gov/clinvar/variation/1683119/dbSNP: rs372255666 P30 — PRKDC c.1655 C>G ClinVar (Variation): https://www.ncbi.nlm.nih.gov/clinvar/variation/992552/dbSNP: rs373088662.

## Abbreviations

AICs: Autoimmune cytopenias.
IEIs: Inborn errors of immunity.
AIHA: Autoimmune hemolytic anemia.
ITP: Immune thrombocytopenia.
AIN: Autoimmune neutropenia.
IVIG: Intravenous immunoglobulin.
NGS: Next-generation sequencing.
CVID: Common variable immunodeficiency.
*TACI*: Transmembrane activator and CAML interactor (TNFRSF13B).
*LRBA*: Lipopolysaccharide-responsive and beige-like anchor protein.
*PRF1*: Perforin 1.
*CECR1*: Cat Eye Syndrome Chromosome Region, Candidate 1 (ADA2).
*PRKDC*: Protein kinase, DNA-activated, catalytic polypeptide.
*MRTFA*: Myocardin-related transcription factor A.
*PSTPIP1*: Proline-serine-threonine phosphatase-interacting protein 1.
